# Secondary signet ring cell carcinoma of the prostate: a case report

**DOI:** 10.1093/jscr/rjaf204

**Published:** 2025-04-06

**Authors:** You Zhao, Weiping Luo, Jun Nie, Hongxiang Ma, Zihao Li

**Affiliations:** Department of Urology, Liyang People's Hospital, Affiliated Hospital of Kangda College of Nanjing Medical University, No. 70 Jianshe West Road, Liyang 213300, China; Department of Urology, Liyang People's Hospital, Affiliated Hospital of Kangda College of Nanjing Medical University, No. 70 Jianshe West Road, Liyang 213300, China; Department of Urology, Liyang People's Hospital, Affiliated Hospital of Kangda College of Nanjing Medical University, No. 70 Jianshe West Road, Liyang 213300, China; Department of Urology, Liyang People's Hospital, Affiliated Hospital of Kangda College of Nanjing Medical University, No. 70 Jianshe West Road, Liyang 213300, China; Department of Urology, Liyang People's Hospital, Affiliated Hospital of Kangda College of Nanjing Medical University, No. 70 Jianshe West Road, Liyang 213300, China

**Keywords:** signet ring cell, carcinoma, prostate

## Abstract

Signet ring cell carcinoma (SRCC) is a rare, highly malignant tumor primarily arising in the gastrointestinal tract, with secondary prostate involvement exceedingly uncommon. We report a 64-year-old male who underwent total gastrectomy for gastric SRCC in 2020, followed by chemotherapy. In 2024, he presented with urinary symptoms. Imaging and laboratory tests suggested benign prostatic hyperplasia, but pathology revealed secondary prostate SRCC, confirmed immunohistochemically. The patient continued gastric cancer chemotherapy and is under follow-up. This case underscores diagnostic and therapeutic challenges, emphasizing the need for histopathological and immunohistochemical exams and further research.

## Introduction

Signet ring cell carcinoma (SRCC) is a unique malignant tumor characterized by its histological features, where mucin-filled cells with nuclei pushed to one side form a signet ring-like structure. This type of carcinoma predominantly arises in the gastrointestinal tract [1]. However, secondary signet ring cell carcinoma of the prostate is exceptionally rare, with only a handful of reports globally, and it is recognized as a highly malignant subtype of prostate cancer with poor prognostic outcomes [2]. Herein, we present a case treated at our hospital in November 2024, along with a comprehensive literature review.

## Case presentation

In June 2020, a 64-year-old male patient underwent total gastrectomy due to a malignant gastric tumor. Intraoperative findings revealed scattered metastatic foci on the round ligament of the liver. The tumor, located in the lesser curvature of the stomach, measured ⁓6 × 5 × 3 cm. It had invaded beyond the serosa, presenting with a hard texture, irregular morphology, poorly defined borders, and infiltrative ulcerative growth. Multiple enlarged lymph nodes were palpable surrounding the stomach. Further exploration showed no metastatic nodules in the liver, pelvis, or abdomen. The surgery was uncomplicated, and the patient recovered well postoperatively. Pathological results indicated that the gastric body harbored signet ring cell carcinoma with mucinous adenocarcinoma, diffusely infiltrating and measuring 14 × 12 × 1.5 cm, with serosal invasion. Cancer metastasis was detected in small curvature lymph nodes (1/9) and large curvature lymph nodes (3/5). Cancerous tissue infiltration was observed in the pancreatic capsule and omental nodules. All other assessed margins and lymph node groups were negative for cancer. Immunohistochemical analysis confirmed gastric body signet ring cell carcinoma with mucinous adenocarcinoma. Tumor cells exhibited positivity for CK7, negativity for CK20 and P53, a Ki-67 proliferation index of 25%, HER-2(0), GPC-3(−), villin(+), and PD-L1(−). The pathological stage was T4N2M1. Postoperatively, the patient received six cycles of chemotherapy with the regimen of “albumin-bound paclitaxel 300 mg d1 + tegafur/gimeracil/oteracil potassium 50 mg bid*14d po Q3W.” Subsequent gastroscopy, abdominal computed tomography (CT), and tumor marker tests demonstrated no significant abnormalities.

**Figure 1:**
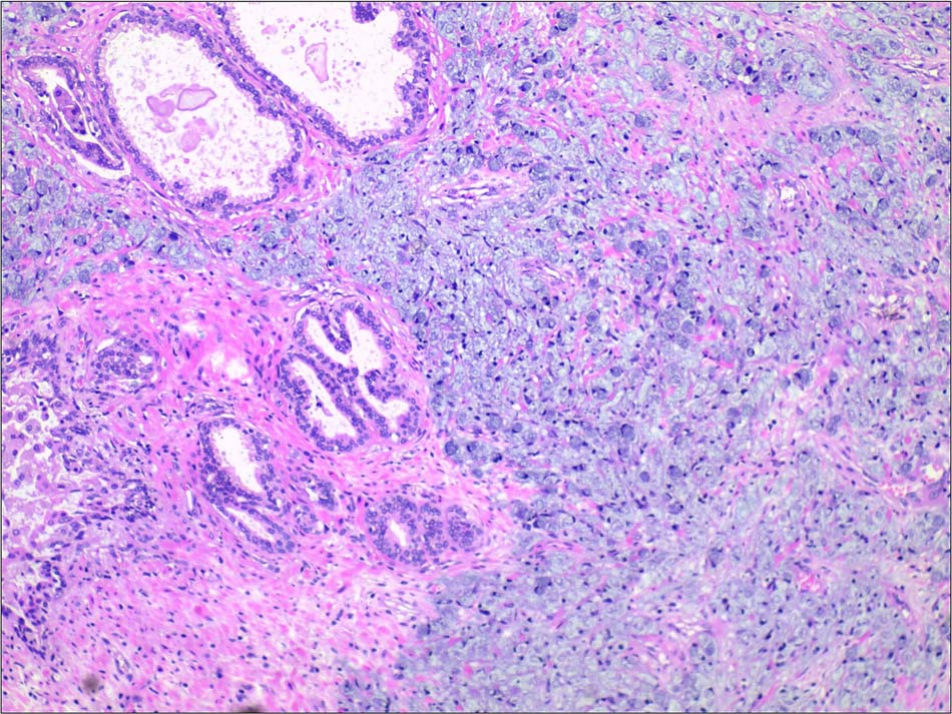
Histopathological examination revealed extensive infiltration of signet-ring cells, with the cytoplasm filled with mucinous vacuoles, and the nuclei showing eccentric positioning and dark staining. H&E stain 40×.

**Figure 2:**
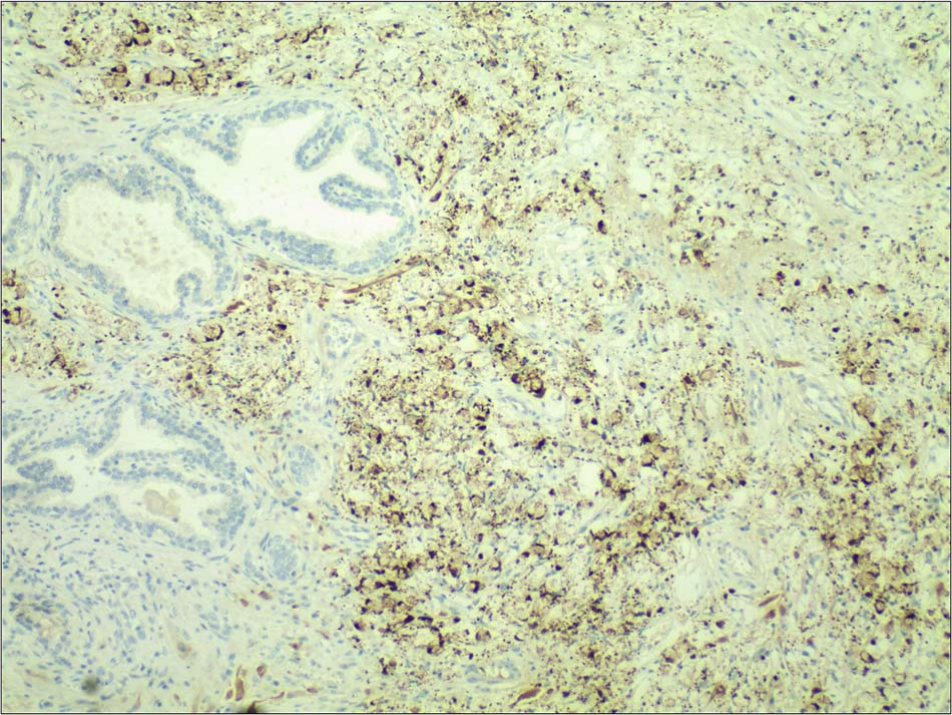
Immunohistochemical staining demonstrated strong positivity (++) for villin in the cytoplasm and cell membrane of tumor cells. IHC stain 40×.

In November 2024, the patient presented with progressive dysuria for 3 years, accompanied by recurrent urinary retention for 3 weeks. Physical examination revealed a prostate enlarged to grade II, with a firm texture, smooth surface, no palpable nodules, no tenderness, a shallow median sulcus, and no sphincter relaxation. Imaging studies with 3.0 T prostate magnetic resonance imaging (MRI) showed a prostate measuring ⁓5.7 × 4.8 × 5.7 cm, with marked enlargement of the transition zone and heterogeneous signals. Small, patchy short T2 signals were internally observed, along with slightly high diffusion-weighted imaging (DWI) signals and slightly decreased apparent diffusion coefficient (ADC) signals, yielding a PI-RADS score of 3. The peripheral zone was thinned and indistinct. The seminal vesicles showed normal size and morphology, and the bladder-seminal vesicle triangle was intact. No enlarged lymph nodes were evident in the pelvic cavity. Laboratory tests showed a total prostate-specific antigen level of 1.759 ng/ml. Initially diagnosed with benign prostatic hyperplasia, the patient underwent transurethral enucleation of the prostate with a thulium laser under general anesthesia on 19 November 2024. The surgery was successful. Postoperative pathology indicated poorly differentiated adenocarcinoma, predominantly signet ring cell carcinoma, presumed to be of gastric origin (Fig. 1). Immunohistochemical analysis revealed negativity for prostate cancer markers (PSA and P504S) and positivity for gastrointestinal tumor markers [CKP(3+), CK20(+), CEA(3+), Villin(2+), CDX-2(2+)] (Fig. 2). Morphological comparison with previous gastric cancer pathology slides showed similar features, confirming metastatic gastric signet ring cell carcinoma to the prostate. The patient continued chemotherapy with the original gastric cancer regimen postoperatively and is currently under follow-up.

## Discussion

Acinar adenocarcinoma accounts for over 90% of prostate cancers, representing the most common pathological type. Among acinar adenocarcinomas, rare histological variants exist, including signet ring cell type, atrophic type, pseudohyperplastic type, foamy gland type, eosinophilic type, and sarcomatoid type [3]. Notably, prostate signet ring cell carcinoma is an extremely rare, highly malignant, rapidly progressing, and poorly prognostic subtype of prostate cancer [4]. Recently, reports on primary prostate signet ring cell carcinoma have increased, drawing considerable clinical attention [5]. Conversely, the incidence of secondary prostate signet ring cell carcinoma is even scarcer. Since Watson *et al*. first reported a case of gastric signet ring cell carcinoma with subsequent prostate metastasis in 1990, pertinent literature has been sparse [6].

Most cases of secondary prostate cancer arise from direct infiltration by tumors in adjacent organs, with only ⁓1% resulting from distant primary cancers other than leukemia or lymphoma [7]. In this case, the most probable route of metastasis from gastric cancer to the prostate is through a specific hematogenous spread [8]. The diagnosis and treatment of secondary prostate signet ring cell carcinoma pose significant challenges [9]. Due to its rarity, clinical awareness of this prostate cancer subtype is insufficient, potentially leading to misdiagnosis or missed diagnosis. Diagnostic accuracy currently relies on histopathological and immunohistochemical examinations, coupled with a history of related tumors [10]. In this case, histopathological examination of the prostate tissue revealed signet ring cell carcinoma, showing morphological similarities to previous gastric cancer pathology slides. Further immunohistochemical analysis confirmed the absence of prostate cancer markers (PSA and P504S) and the presence of gastrointestinal tumor markers [CKP(3+), CK20(+), CEA(3+), Villin(2+), CDX-2(2+)], definitively diagnosing the case as secondary prostate signet ring cell carcinoma.

Regarding treatment, secondary signet ring cell carcinoma of the prostate may exhibit treatment resistance, particularly to endocrine therapy, which is often ineffective. Studies suggest that radical prostatectomy combined with pelvic lymph node dissection and postoperative radiotherapy could be a reasonable option [9]. However, according to the fundamental principles of oncology, chemotherapy targeting the primary gastric cancer should be the mainstay of treatment [11]. For patients with severe lower urinary tract obstruction symptoms, transurethral prostatectomy may be considered to alleviate symptoms, based on the patient’s physical condition. In this case, following surgery for benign prostatic hyperplasia, the patient’s lower urinary tract obstruction symptoms improved substantially. Upon postoperative diagnosis of secondary prostate signet ring cell carcinoma, the patient received further chemotherapy targeting the primary gastric cancer regimen, achieving a satisfactory prognosis to date.

In conclusion, secondary prostate signet ring cell carcinoma, as an exceedingly rare subtype of prostate cancer, lacks definitive conclusions regarding its diagnosis and treatment, necessitating further research and exploration in the future.

## Data Availability

Data sharing is available upon reasonable request from the corresponding author.
